# Increasing co-occurrence of fine particulate matter and ground-level ozone extremes in the western United States

**DOI:** 10.1126/sciadv.abi9386

**Published:** 2022-01-05

**Authors:** Dmitri A. Kalashnikov, Jordan L. Schnell, John T. Abatzoglou, Daniel L. Swain, Deepti Singh

**Affiliations:** 1 School of the Environment, Washington State University Vancouver, Vancouver, WA, USA.; 2 Cooperative Institute for Research in Environmental Sciences, University of Colorado Boulder, NOAA/Global Systems Laboratory, Boulder, CO, USA.; 3 Management of Complex Systems Department, University of California, Merced, Merced, CA, USA.; 4 Institute of the Environment and Sustainability, University of California, Los Angeles, Los Angeles, CA, USA.; 5 Capacity Center for Climate and Weather Extremes, National Center for Atmospheric Research, Boulder, CO, USA.; 6 The Nature Conservancy of California, San Francisco, CA, USA.

## Abstract

Wildfires and meteorological conditions influence the co-occurrence of multiple harmful air pollutants including fine particulate matter (PM_2.5_) and ground-level ozone. We examine the spatiotemporal characteristics of PM_2.5_/ozone co-occurrences and associated population exposure in the western United States (US). The frequency, spatial extent, and temporal persistence of extreme PM_2.5_/ozone co-occurrences have increased significantly between 2001 and 2020, increasing annual population exposure to multiple harmful air pollutants by ~25 million person-days/year. Using a clustering methodology to characterize daily weather patterns, we identify significant increases in atmospheric ridging patterns conducive to widespread PM_2.5_/ozone co-occurrences and population exposure. We further link the spatial extent of co-occurrence to the extent of extreme heat and wildfires. Our results suggest an increasing potential for co-occurring air pollution episodes in the western US with continued climate change.

## Teaser

More frequent atmospheric ridging and wildfires increase exposure of western U.S. population to co-occurring air pollutants.

## INTRODUCTION

Air pollution is an urgent global health problem, and one that has gained additional attention during the coronavirus disease 2019 (COVID-19) pandemic due to the exacerbating effects of pollutant exposure on infectious disease spread and mortality (*1*–*3*). Two main air pollutants—fine particulate matter (PM_2.5_, defined as particulate matter with diameter of ≤2.5 μm) and ground-level ozone (hereafter, “ozone”)—are linked to significant human health concerns including cardiovascular and respiratory illnesses and mortality (*4*–*7*). PM_2.5_ and ozone have also been linked to negative ecosystem impacts via their detrimental effects on plants and the broader environment (*8*–*10*). Although few studies have quantified the compounding health impacts of co-occurring PM_2.5_ and ozone, existing research indicates that simultaneous exposure to both pollutants can have disproportionately more severe health impacts beyond the individual effect of either pollutant (*11*, *12*).

Wildfires can cause simultaneous increases in both pollutants through the direct emission of PM_2.5_ (*13*, *14*) and ozone precursor compounds (*15*–*18*) in smoke plumes, and recent research has shown that ozone concentrations in urban areas in the western United States (US) can be enhanced in the presence of wildfire smoke (*19*–*21*). During years of limited wildfire activity, most of the western US experienced annual maximum PM_2.5_ concentrations during the cool season when stagnant air conditions are typically prevalent (*22*). This seasonality would typically minimize co-occurrence risk with high ozone concentrations, which peak during the warm season when hot and dry conditions facilitate the formation and build-up of ozone (*23*). Summertime wildfires therefore present a mechanism for PM_2.5_ extremes to occur at a time of year when ozone levels are seasonally high, leading to increased chances of elevated concentrations of both air pollutants occurring simultaneously.

Smoke from the unprecedented wildfire activity in the western US during August and September of 2020 contributed to several weeks of extremely hazardous air quality over a large area (*3*, *24*). Similar conditions, although on a smaller scale, occurred during the 2015, 2017, and 2018 wildfire seasons (*25*, *26*), and extensive wildfire smoke affected the region again in 2021. These widespread hazardous conditions acutely affected vulnerable communities in the region—those at enhanced risk due to socio-economic or demographic factors and underlying health conditions—contributing to an increased burden on the health care system through increased hospitalizations and emergency department visits (*26*). In addition, recent research has linked wildfire smoke in 2020 to higher risk and mortality associated with COVID-19 in many western US states (*3*). As recurrent and prolonged exposure to air pollution can exacerbate the public health impacts of wildfire smoke (*27*–*31*), recent wildfire seasons have thus raised significant concerns regarding the trajectory of air quality in the region.

Historical and projected climate and wildfire trends in the western US both point toward increasing risk of exposure to poor air quality. Increased wildfire activity has already contributed to rising extreme PM_2.5_ concentrations in fire-prone regions of the western US (*32*, *33*), offsetting national-level air quality improvements following the Clean Air Act. Wildfires have contributed up to 50% of annual PM_2.5_ in parts of the western US in recent years (*34*). Annual burned area across the western US has experienced exponential growth in recent decades (*35*, *36*), partially due to drying of vegetation in the region tied to anthropogenic climate change (*37*–*39*). These observed trends are projected to continue in a warming climate (*24*, *40*–*42*).

Long-term climate and daily-scale meteorological conditions both influence the formation, accumulation, and transport of air pollutants. Large-scale high-pressure systems (or “ridges”) during the summer enhance surface temperatures, promote air stagnation, and can contribute to both increased wildfire activity and ozone production in the western US (*23*). Previous work has shown that these high-pressure systems are expected to increase in frequency and persistence due to climate change (*43*, *44*), raising the potential for increased warm-season co-occurrence of PM_2.5_/ozone extremes in the future. These conditions are amplified in the western US by topography that promotes air stagnation in populated regions adjacent to fire-prone lands (e.g., the Los Angeles Basin and the Willamette Valley near Portland).

Despite rising public health and air quality concerns, the influence of increasing wildfire activity and changing meteorology on widespread hazardous air quality conditions across the geographic extent of the western US has not yet been investigated. Schnell and Prather (*45*) systematically demonstrated the influence of meteorology on the co-occurrence of PM_2.5_, ozone, and temperature extremes over eastern North America. Western North America, however, has fundamentally different seasonality and drivers of these pollutants, and previous studies investigating air pollutant co-occurrences have been restricted to urban areas [e.g., (*19*–*21*)]. Understanding how regional factors influence air pollutant characteristics and contribute to their changing risks is critical for assessing their public health impacts and anticipating future trends associated with climate variability and change.

Given the compounding human health impacts of air pollutant co-occurrences, we investigate the influence of wildfires and meteorological factors on the spatial and temporal characteristics of extreme PM_2.5_/ozone co-occurrences across the western US and assess the associated population exposure. Using gridded 1° × 1° datasets of observed PM_2.5_ and ozone developed by Schnell *et al.* (*46*) and atmospheric reanalyses, we (i) quantify trends in the frequency, persistence, and extent of widespread co-occurrence of PM_2.5_/ozone extremes across the western US in the past two decades; (ii) identify the large-scale atmospheric patterns associated with widespread co-occurrences and population exposure; (iii) examine trends in atmospheric patterns that amplify or mitigate co-occurrence risk across the region; and (iv) investigate the relationship between the geographic extent of co-occurrence, wildfire activity, and extreme heat during and preceding widespread PM_2.5_/ozone co-occurrences. We also investigate these factors in the context of the exceptional widespread and long-lasting co-occurrence episode during the record-breaking 2020 wildfire season.

## RESULTS

### Increasing trends in the spatial and temporal characteristics of PM_2.5_/ozone co-occurrence

**Fig. 1.**
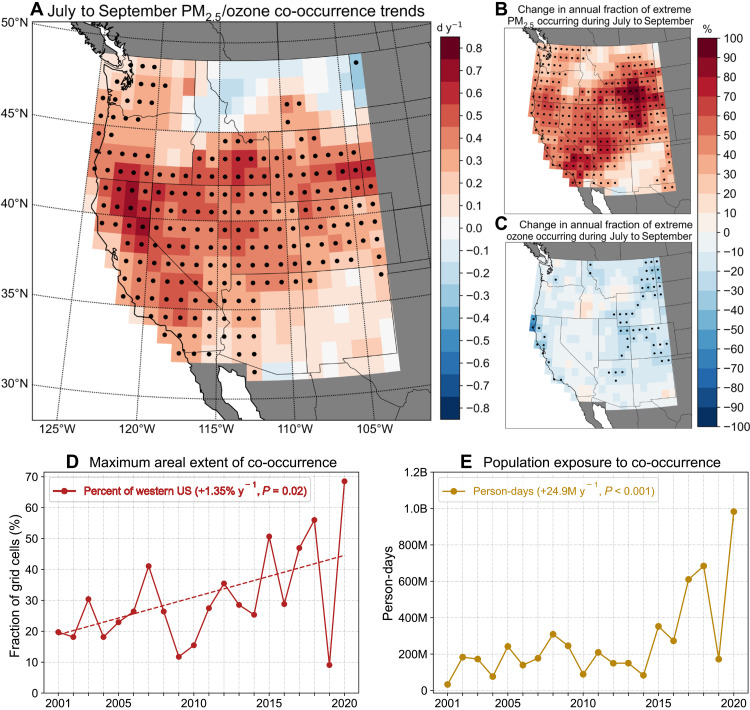
PM_2.5_/ozone co-occurrence trends during 2001 to 2020 and population exposure. (**A**) Trends in the number of days (d y^−1^) with PM_2.5_/ozone co-occurrences at each grid cell during July to September. Co-occurrences are defined as values of each pollutant exceeding their respective local annual 90th percentile daily concentrations simultaneously. Trends in the annual fraction of (**B**) PM_2.5_ extremes and (**C**) ozone extremes occurring at each grid cell during July to September relative to rest of year (October to June). The maximum possible number of co-occurrences is 37 per year in each grid cell, equal to the number of days above local annual 90th percentile daily concentration values for each pollutant. Black dots denote statistical significance of trends at *P* < 0.05 based on a nonparametric permutation test. (**D**) Maximum daily extent of western US grid cells simultaneously experiencing co-occurrences of local PM_2.5_/ozone extremes during July to September each year. (**E**) Total population exposure to all local PM_2.5_/ozone co-occurrences during July to September measured in million person-days (M) per year. Text in (D) and (E) indicates the linear trends and *P* values based on a permutation test.

Extremes in individual air pollutant concentrations are defined at each grid cell as exceedances of their annual 90th percentiles (~37 days each year). We find that the simultaneous, spatially colocated occurrence of local PM_2.5_ and ozone extremes (hereafter “co-occurrence”) has become significantly more frequent over large areas of the western US during the late-summer wildfire season—July to September—between 2001 and 2020, driven largely by the changing seasonality of extreme PM_2.5_ concentrations (Fig. 1). High PM_2.5_ concentrations typically peaked during cool-season months across much of this region during the early 2000s (fig. S1A). However, the fraction of the annual PM_2.5_ extremes occurring during July to September has increased significantly in the past two decades (Fig. 1B). Parts of the region experienced a >80% increase in this fraction, indicating that, in these grid cells, most of PM_2.5_ extremes are now concentrated during this season that previously rarely experienced PM_2.5_ extremes. In contrast, ozone concentrations typically peak during warm-season months (fig. S1, C and D), and the fraction of annual ozone extremes occurring during July to September remains largely unchanged with the exception of small decreases over parts of the Rocky Mountains, High Plains, and coastal California (Fig. 1C). Therefore, the increased occurrence of PM_2.5_ extremes during a time of year when ozone concentrations are seasonally high has largely driven the observed increases in PM_2.5_/ozone co-occurrence during the late-summer wildfire season across the western US (Fig. 1A).

In addition to occurring more frequently, local PM_2.5_/ozone co-occurrences are increasingly occurring across a larger geographic region simultaneously. The maximum daily fraction of western US grid cells with simultaneous PM_2.5_/ozone co-occurrence during July to September has more than doubled (from 18.9 to 44.6%) over the past two decades, with an increasing trend of ~1.35% per year (*P* = 0.02) (Fig. 1D). The largest spatial extents of co-occurrence were observed in 2015, 2017, 2018, and 2020—coincident with hot, dry summers and widespread fire activity, including the largest burned areas across the western US wildland-urban interface (*20*, *24*, *25*, *29*, *36*, *47*). Increases in the frequency and spatial extent of co-occurrences are associated with an increasing trend in July to September population exposure of ~24.9 million person-days/year (*P* < 0.001) in the western US during 2001 to 2020 (Fig. 1E). Cumulative population exposure over the season to PM_2.5_/ozone co-occurrences exceeded 600 million person-days during the 2017, 2018, and 2020 wildfire seasons (Fig. 1E). Daily population exposure exceeded 35 million people during the most widespread air pollution conditions in these three seasons, peaking at ~46 million people (>50% of the western US population) on 21 August 2020 (table S1).

**Fig. 2.**
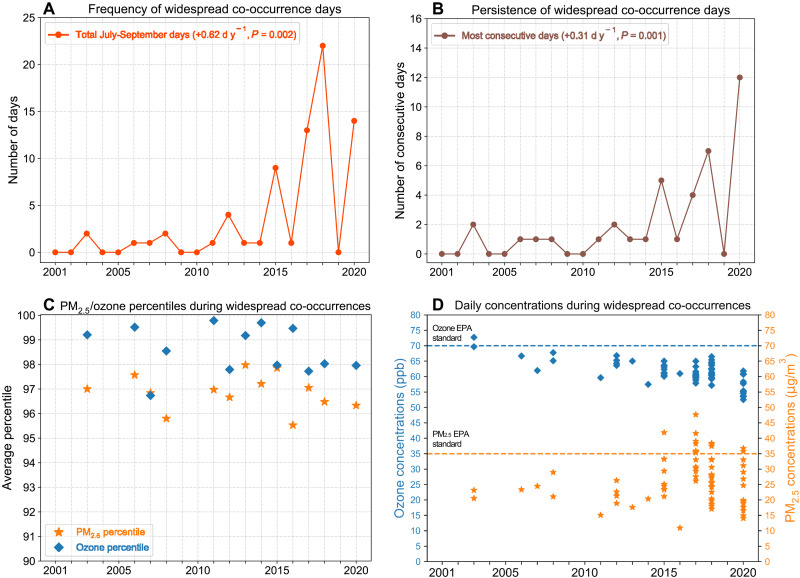
Widespread PM_2.5_/ozone co-occurrences. Time series of (**A**) the total number and (**B**) longest consecutive-day persistence of widespread July to September co-occurrence days, defined as days with simultaneous local PM_2.5_/ozone co-occurrence in ≥25% of western US grid cells. Text in (A) and (B) indicates linear trends (d y^−1^) with *P* values based on a permutation test. Characteristics of the individual pollutants during widespread co-occurrences are shown through (**C**) percentiles of PM_2.5_ and ozone daily concentrations averaged across all affected grid cells and (**D**) pollutant concentrations averaged across affected grid cells on widespread co-occurrence days (*n* = 72). Note that percentiles in (C) are calculated on the basis of the distribution of concentrations in each year (refer to Materials and Methods). Dashed lines in (D) show concentrations corresponding to the Environmental Protection Agency (EPA) regulatory health standards for each pollutant [70 parts per billion (ppb) for ozone and 35 μg/m^**3**^ for PM_2.5_].

Widespread PM_2.5_/ozone co-occurrences, defined as days on which at least 25% of grid cells covering the western US simultaneously experience local PM_2.5_/ozone co-occurrence, have occurred almost exclusively during July to September (72 of 75 total days; fig. S2). Widespread co-occurrences have become significantly more frequent and persistent (Fig. 2, A and B), with an increase of ~12.4 widespread co-occurrence days over 2001 to 2020 and the longest consecutive-day occurrence persisting for an additional ~6.2 days. The frequency of widespread co-occurrences was highest during the recent active wildfire seasons (Fig. 2A and fig. S3). Of the 72 July to September widespread co-occurrence days during 2001 to 2020, 59 occurred during 2015, 2017, 2018, and 2020. In addition, the longest persistence (12 consecutive days) of widespread PM_2.5_/ozone co-occurrences on record occurred in 2020 (Fig. 2B), during which the daily maximum extent of co-occurrence peaked at ~68.5% of the western US on 24 August 2020 (Fig. 1D and table S1).

During widespread co-occurrences, the concentrations of both pollutants are elevated relative to co-occurrence conditions of smaller geographic extent. Although co-occurrences are defined as values above the local, annual 90th percentiles for both PM_2.5_ and ozone in each grid cell, average observed concentrations on all widespread co-occurrence days exceeded the 95th percentile for PM_2.5_ and the ~97th percentile for ozone across all grid cells experiencing local PM_2.5_/ozone co-occurrence (Fig. 2C). These findings are consistent with those of Schnell and Prather (*45*), who reported enhancements in PM_2.5_ and ozone concentrations over eastern North America during large, multiday pollution episodes well above the statistical thresholds used to define individual extremes (e.g., 90th or 95th percentile). During widespread co-occurrence days in recent seasons (2015, 2017, 2018, and 2020), PM_2.5_ concentrations averaged across all constituent grid cells experiencing PM_2.5_/ozone co-occurrence exceeded the Environmental Protection Agency (EPA) regulatory limit of 35 μg/m^3^ on 13 individual days (Fig. 2D, orange markers), peaking at 47.7 μg/m^3^ on 3 September 2017 during a period of widespread fire activity and smoke conditions in the western US (*25*). Ozone concentrations averaged across the same grid cells on these days (*n* = 13) ranged from 57 to 63 parts per billion (ppb) (Fig. 2D, blue markers; see also fig. S4 for average concentrations during all co-occurrences). Although below the EPA regulatory limit of 70 ppb, the fact that these high ozone concentrations were present when averaged over a large geographic area and for prolonged periods in combination with widespread PM_2.5_ regulatory exceedances illustrates the magnitude of human and environmental exposure to harmful air pollutants during recent wildfire seasons, the health impacts of which are emerging (*3*, *26*, *30*, *31*).

### Increasing trends in atmospheric patterns conducive to co-occurrence

Although wildfires are a key source of emissions of PM_2.5_ and ozone precursor compounds during the late-summer season, the spatial extent, local concentrations, and temporal persistence of their co-occurrences are modulated by a suite of meteorological factors, including surface temperature and atmospheric patterns (*45*). To understand whether and how atmospheric patterns that affect PM_2.5_/ozone co-occurrence characteristics are changing, we use a spatial clustering approach known as self-organizing maps (SOMs) (*48*, *49*). Our SOM implementation categorizes daily large-scale weather patterns during July to September into 12 representative clusters (or “nodes”) based on 500-hPa geopotential height anomalies from the European Centre for Medium-Range Weather Forecasts (ECMWF) ERA5 reanalysis product (1979 to 2020; refer to Materials and Methods).

**Fig. 3.**
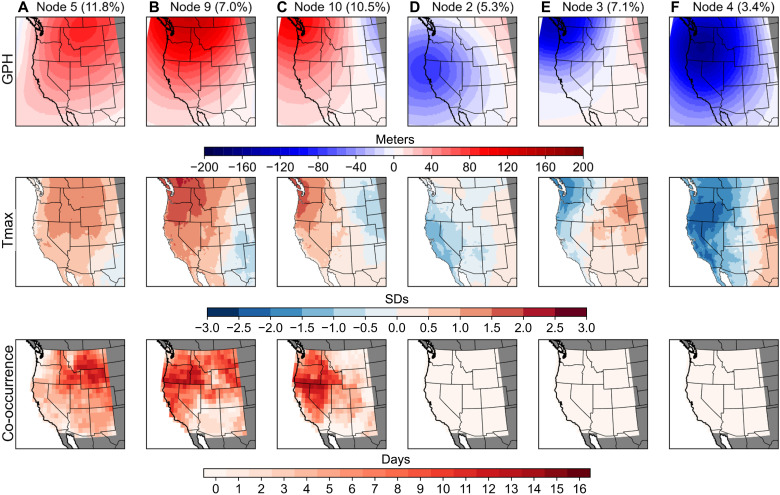
The six SOM nodes with the largest [(A) to (C)] and smallest [(D) to (F)] PM_2.5_/ozone co-occurrence risk. Top: Geopotential height (GPH) anomalies for each SOM node trained over 1979 to 2020. Middle: Composite standardized anomalies of daily maximum temperatures (T_max_) on all days associated with each node during the 2001 to 2020 period. Bottom: Number of times each grid cell experienced local PM_2.5_/ozone co-occurrences during all widespread co-occurrence days associated with that node. The maximum possible number of co-occurrence days in a given grid cell is equivalent to the total number of widespread co-occurrence days associated with that node (node 5, 14 days; node 9, 16 days; node 10, 13 days; Table 1). Values in parentheses on top row indicate the frequency of each SOM node relative to all July to September days during the period of overlap with air pollution data (2001 to 2020).

We quantify the number of widespread co-occurrence days and population exposure to co-occurrence associated with each node (Table 1 and fig. S5) and identify the six SOM nodes with the largest (nodes 5, 9, and 10; hereafter, “high-exposure nodes”) and smallest (nodes 2, 3, and 4; hereafter, “low-exposure nodes”) PM_2.5_/ozone co-occurrence risk (Fig. 3). High-exposure nodes are characterized by widespread positive geopotential height anomalies (hereafter, “ridging”) and high daily maximum surface temperature anomalies across the region, which are largely colocated with those grid cells experiencing the highest number of local PM_2.5_/ozone co-occurrences during widespread co-occurrence days in that node (Fig. 3, A to C). In contrast, low-exposure nodes are characterized by widespread anomalously low geopotential heights, cooler temperatures, and onshore airflow from the Pacific Ocean, providing critical natural ventilation for this region and suppressing widespread co-occurrence risk (Fig. 3, D to F) (*50*).

**Table 1.** Summary statistics for all 12 nodes of the self-organizing map (SOM). PM_2.5_/ozone co-occurrence data represents all July to September days from 2001 to 2020.

**SOM node**	**Number of days**	**Cumulative PM_2.5_./ozone** **co-occurrence exposure** **in million person-days**	**Number of widespread** **PM_2.5_/ozone co-occurrence** **days (*n* = 72)**	**42-year change in** **SOM node frequency** **(days/year)**	**42-year change in maximum** **SOM node persistence** **(consecutive days/year)**
**1**	151	441.7	5	−1.1	−0.6
**2**	98	78.2	0	−6.4*	−3.5*
**3**	130	77.9	0	−2.0	−0.2
**4**	63	4.4	0	−4.2*	−1.7
**5**	217	835.7	14	5.8*	1.2
**6**	198	529.2	6	0.0	−0.1
**7**	251	650.8	8	2.7	0.7
**8**	110	122.6	2	−2.2	−1.0
**9**	129	866.7	16	2.2	0.6
**10**	194	847.2	13	6.2*	2.1*
**11**	179	739.5	8	2.8	0.6
**12**	120	141.0	0	−3.7	−0.7

*Statistical significance of node trends (1979 to 2020) at *P* < 0.05.

**Fig. 4.**
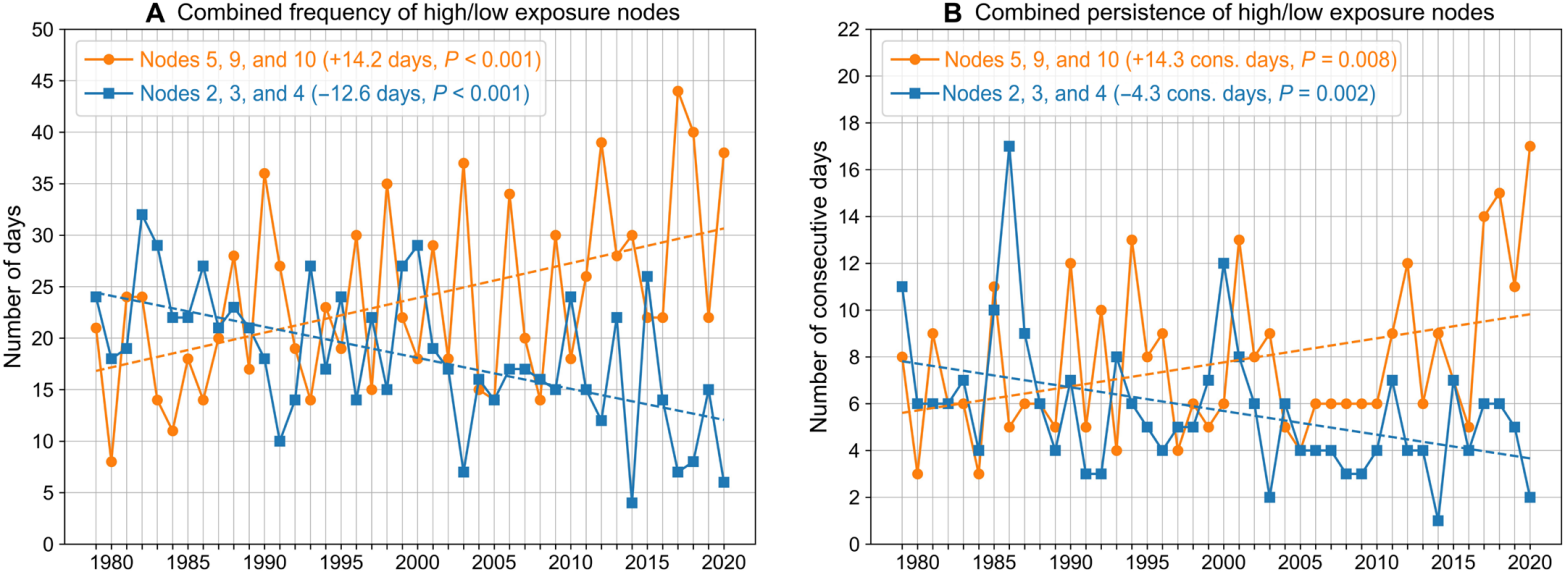
Frequency and persistence of high/low exposure nodes. Time series of combined (**A**) total number of days and (**B**) longest multiday persistence for high-exposure SOM nodes 5, 9, and 10 (orange lines) and low-exposure SOM nodes 2, 3, and 4 (blue lines), during July to September, 1979 to 2020. In both plots, dashed lines show linear trends with numbers indicating corresponding changes over the 42-year period and *P* values of the linear trends based on a permutation test.

Large-scale atmospheric patterns represented by high-exposure nodes contributed 43 of the 72 widespread co-occurrence days (~60%), despite accounting for only ~29% of all July to September days since 2001, indicating an elevated risk of PM_2.5_/ozone co-occurrence across the region when these patterns occur. We find robust increases in the frequency and persistence of high-exposure nodes since 1979. These nodes now occur on an additional ~14.2 days/ year during July to September (*P* < 0.001) and the longest persistence of these nodes is an additional ~4.3 consecutive days longer (*P* = 0.008) compared to four decades ago (Fig. 4, orange lines). While the frequency of nodes relates to the frequency of pollutant exposure, the longer persistence of certain nodes can have additional impacts beyond that of single-day node occurrences. For example, previous research has shown that high ozone concentrations are more likely during prolonged, multiday heat conditions than on single hot days (*22*, *45*). Of the 29 remaining widespread co-occurrence days not associated with the high-exposure nodes, 21 occurred in conjunction with atmospheric patterns favorable for widespread smoke transport across the region during periods of high wildfire activity (nodes 1, 7 and 11; see Table 1 and fig. S5). In contrast to the high-exposure nodes, the combined frequency and multiday persistence of low-exposure nodes exhibit negative trends during 1979 to 2020, now occurring on ~12.6 fewer days/year (*P* < 0.001) and the longest consecutive-day occurrence of these nodes persisting for ~4.3 fewer days (*P* = 0.002) compared to four decades ago (Fig. 4, blue lines).

Together, these results suggest that atmospheric patterns that are conducive to widespread local PM_2.5_/ozone co-occurrences and larger population exposure across the western US are becoming more frequent and persistent during July to September. Recent active wildfire seasons have occurred in conjunction with record frequency and persistence of the high-exposure nodes (i.e., ridging), with the highest frequency since 1979 of 44 days observed in July to September 2017 and longest persistence of 17 consecutive days observed from 3 to 19 September 2020 occurring simultaneously with historic wildfire activity across several western US states (Fig. 4) (*24*). The observed increase in ridging has co-occurred with and likely amplified increasing aridity and extent of wildfire burned area over the western US at least partially associated with anthropogenic warming, posing compounding hazards to the region (*37*–*39*, *51*). Furthermore, increased persistence of ridging during wildfire smoke conditions can exacerbate ground-level pollution in topographically constrained basins, as decreased sunlight and increased atmospheric stability trap smoke and prolong the air pollution conditions (*52*, *53*). Conversely, atmospheric patterns favoring decreased widespread PM_2.5_/ozone co-occurrences across the western US (i.e., negative geopotential height anomalies and onshore airflow) are appearing less often and with shorter duration during the late-summer wildfire season.

### Case study: Widespread co-occurrence episode of August 2020

**Fig. 5.**
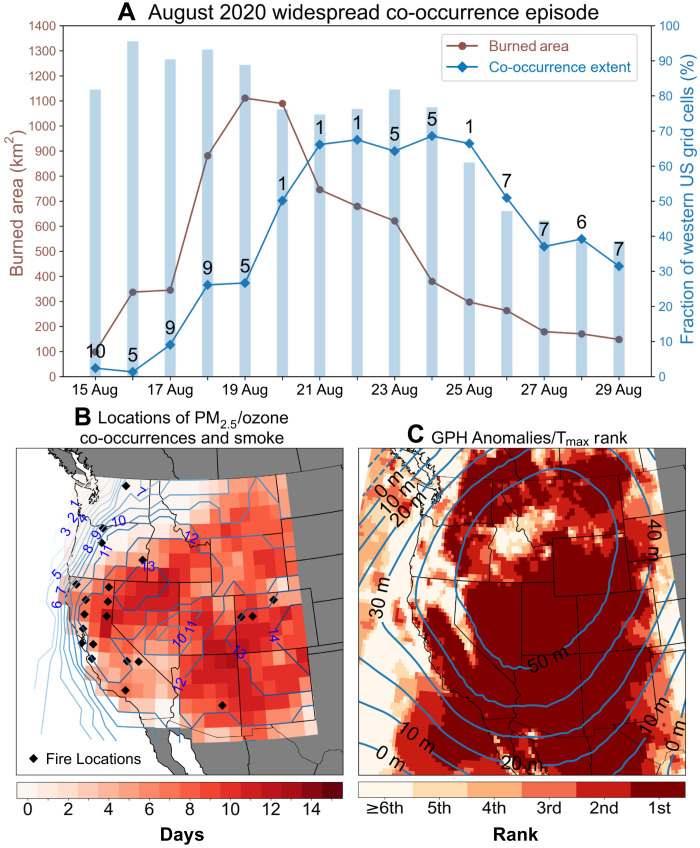
Widespread co-occurrence episode during 15 to 29 August 2020. (**A**) Time series of daily burned area from MODIS in the western US and southwest Canada (brown line), fraction of western US grid cells with local PM_2.5_/ozone co-occurrence (blue line), and fraction of western US grid cells with T_max_ anomalies exceeding 1 SD above local daily climatology (blue bars). Numbers on the blue line indicate the best-matching SOM node for that day’s atmospheric pattern. Note that widespread co-occurrence conditions begin on 18 August and persist through 29 August. (**B**) Total number of local PM_2.5_/ozone co-occurrence days at each grid cell (shaded) and total number of days with presence of smoke from National Oceanic and Atmospheric Administration’s (NOAA’s) Hazard Mapping System (HMS) (contours) between 15 and 29 August. Black markers indicate presence of wildfires from MODIS in at least 50 1-km grid cells contained within each of the 1° × 1° grid cells of the PM_2.5_/ozone data. (**C**) Average GPH anomalies (contours every 10 m; as in Fig. 3, top row) and rank of the average T_max_ during 15 to 29 August 2020 compared to all other similar 2-week periods during 1979 to 2019 (shaded). The darkest red shading indicates that in 2020 those grid cells experienced their hottest average T_max_ within the 42-year ERA5 dataset.

The “exceptional” 2020 wildfire season featured the second highest number of widespread PM_2.5_/ozone co-occurrence days across the domain, along with the longest consecutive-day persistence of widespread co-occurrence (Fig. 2, A and B), the single most widespread daily co-occurrence extent (~68.5%) across the western US (Fig. 1D) and the highest cumulative seasonal population exposure to all local PM_2.5_/ozone co-occurrences of nearly 1 billion person-days (Fig. 1E) in the 20-year observed record. Widespread wildfire activity and extreme temperatures associated with atmospheric ridging both contributed to shaping the record multiday co-occurrence episode observed during the second half of August 2020 (Fig. 5).

To examine their influence in shaping the multiday widespread air pollution episode, we analyze the wildfire and meteorological conditions between 15 and 29 August 2020. We find a sharp increase in the spatial extent of locally defined PM_2.5_/ozone co-occurrences immediately following a peak in daily burned area aggregated over the western US and southwest Canada (Fig. 5A, brown line). This increase in burned area was associated with an extremely anomalous dry lightning outbreak that ignited hundreds of wildfires, leading to multiple large fires that burned for several weeks in central and northern California (Fig. 5B) (*24*). Grid cells in large areas of the interior western US, both near and downwind of fires, observed PM_2.5_/ozone co-occurrences on most of the days (>7) during this 15-day period (Fig. 5B, shading). The grid cells that experienced a high number of co-occurrences are largely colocated with areas where wildfire smoke persisted during that period, which is identified by the National Oceanic and Atmospheric Administration’s (NOAA’s) Hazard Mapping System (HMS) smoke product (Fig. 5B, contours). Notably, grid cells in northern Nevada immediately downwind of California fires observed local PM_2.5_/ozone co-occurrences on at least 12 days, and smoke was observed on at least 13 days of the 15-day episode. In addition, many grid cells in the interior western US observed record warmest 15-day average of daily maximum temperatures since 1979, conditions that likely enhanced ozone production and contributed to the widespread extent of PM_2.5_/ozone co-occurrences (Fig. 5C) (*23*).

Large-scale atmospheric patterns shaped multiple aspects of this air pollution episode, including the high temperatures, wildfires, and smoke transport. Atmospheric ridging across the western US resembling the pattern of the high-exposure nodes contributed to the hot, dry, and stagnant air conditions conducive to wildfire ignition and pollutant accumulation from smoke during the first 5 days of the episode (15 to 19 August) (Fig. 5A). More than 75% of the western US experienced daily maximum temperature anomalies exceeding 1 SD (σ) on all 5 days. Following the large increase in burned area during this time, a shift to an atmospheric pattern characterized by ridging centered in the interior West (node 1, see fig. S5A) developed on 20 August and persisted for 3 days, resulting in southwest-to-northeast atmospheric airflow in the western part of the domain (Fig. 5A). This pattern transported smoke from California fires across large areas of the interior western US, contributing to an increase in local PM_2.5_/ozone co-occurrence extent from <30 to ~66% of the western US grid cells by 21 August (Fig. 5A, blue line).

The remote transport of wildfire smoke containing multiple pollutants including PM_2.5_ and ozone to areas experiencing record warm conditions and enhanced photochemical ozone production (Fig. 5C) were critical to the widespread extent of this episode. Closer to active fires, dense smoke blocks solar radiation and mitigates ozone production (*19*). In addition, previous studies have noted that aged smoke is more conducive to downwind ozone production [e.g., (*16*, *20*, *21*)], promoting local PM_2.5_/ozone co-occurrences in remote areas where smoke is transported. However, the contribution of wildfire smoke to increased ozone concentrations and thus increased PM_2.5_/ozone co-occurrences needs to be further understood. Buysse *et al.* (*19*) found that the presence of wildfire smoke enhances ozone concentrations in urban areas of the western US, particularly in smoke plumes away from fire sources with PM_2.5_ concentrations below 50 μg/m^3^. Similarly, Brey and Fischer (*21*) and Gong *et al.* (*20*) noted general enhancement of ozone concentrations on smoke days in the western US. However, they also noted distinct regional variation with some locations not observing increased ozone concentrations during smoke conditions.

### Relationships between burned area, heat extremes, and PM_2.5_/ozone co-occurrence

**Fig. 6.**
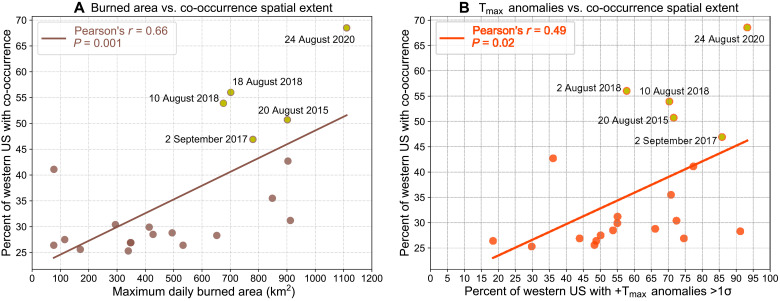
Relationship between widespread PM_2.5_/ozone co-occurrence extent, wildfire burned area, and daily maximum temperatures. Scatterplots showing the spatial extent of the western US affected by PM_2.5_/ozone co-occurrence with (**A**) 7-day lagged MODIS burned area in the western US and southwest Canada and with (**B**) 7-day lagged spatial extent of positive maximum temperature (+T_max_) anomalies >1 SD above local daily climatologies in the western US during the period of overlap with available burned area data (2003 to 2020). For both burned area and +T_max_, the values represent the maximum daily extent in the 7 days preceding the peak spatial extent of PM_2.5_/ozone co-occurrences. Only temporally independent widespread co-occurrence extent peaks during July to September are included (*n* = 21; refer to Materials and Methods). Dates for the top five largest extent peaks are shown. Text in panels indicates Pearson correlation coefficients (*r*) and *P* values for the pairwise relationships.

The dynamics of the August 2020 widespread co-occurrence episode highlight the importance of both meteorology and wildfire extent in shaping the extent of PM_2.5_/ozone co-occurrences and, therefore, exposure. We thus further characterize this relationship between wildfire burned area, meteorology, and the peak spatial extent of all temporally independent widespread co-occurrence periods (*n* = 21; refer to Materials and Methods) (Fig. 6). Given its relevance for ozone production, we specifically focus on relating daily maximum temperature anomalies to PM_2.5_/ozone co-occurrence extent.

Similar to the August 2020 episode, we find that the extent of wildfire activity and heat affect the spatial extent of other PM_2.5_/ozone co-occurrences. The largest extents exceeding 45% of the western US (much larger than the threshold used to define a widespread co-occurrence day) occurred in 2015, 2017, 2018, and 2020 and were associated with extensive wildfire activity in the western US and southwest Canada (maximum daily burned area extent >650 km^2^, Fig. 6A) and widespread positive daily maximum temperature anomalies exceeding 1σ (maximum daily extent ≥55% of the western US, Fig. 6B) in the 7 days preceding the peak spatial extent of temporally independent widespread PM_2.5_/ozone co-occurrence periods (refer to Materials and Methods). Analyzing this relationship over these 21 independent co-occurrence spatial extent peaks during 2003 to 2020, we find robust pairwise correlation between the PM_2.5_/ozone co-occurrence extent and lagged burned area (*r* = 0.66, *P* = 0.001) as well as co-occurrence extent and 7-day lagged extent of anomalously high (>1σ) maximum temperature anomalies (*r* = 0.49, *P* = 0.02). These findings emphasize the role of simultaneous widespread heat and wildfire activity in shaping widespread PM_2.5_/ozone co-occurrences, with high values of this combination of contributing factors in four of the six most recent July to September seasons in the western US.

## DISCUSSION

### Summary

Our analysis demonstrates an increasing risk of exposure of the western US population to more frequent and persistent extreme PM_2.5_/ozone co-occurrences, defined at each grid cell as the simultaneous exceedance of the local annual 90th percentile concentrations of both pollutants, during the late summer wildfire season. These trends are largely driven by PM_2.5_ extremes shifting toward the summer associated with increased wildfire activity in recent years (*24*–*26*, *32*–*39*) and coinciding with the season of high ozone concentrations. PM_2.5_/ozone co-occurrences are also affecting larger areas, with more than a doubling of the maximum daily spatial extent (18.9 to 44.6%) of the western US experiencing simultaneous local co-occurrences over the past two decades. We find that increasing widespread pollutant co-occurrences are associated with increasing wildfire activity and increasing occurrence of conducive atmospheric patterns.

The increase in widespread PM_2.5_/ozone co-occurrences during July to September highlights the role of increasingly severe and larger wildfires in contributing to compounding public health hazards in the western US. Although wildfire smoke can be transported to this region from remote areas including Alaska (*54*) and Siberia (*55*), we find a robust correlation between burned area in the western US and adjacent southwest Canada and extent of local PM_2.5_/ozone co-occurrence across the western US (Fig. 6A). Years with the maximum extent of co-occurrence (Fig. 1D, red line) and greatest frequency of widespread co-occurrence days (Fig. 2A) also experienced the highest mean burned area in the western US [see figure 7 in (*35*)]. The largest spatial extents of co-occurrence in the observed record have all occurred since 2015 during particularly active wildfire seasons, with record co-occurrence extent and persistence in 2020 coinciding with record-breaking wildfire activity in several western US states. Given its ability to produce PM_2.5_ extremes at a time of year when ozone concentrations are seasonally high, our results imply that increasing wildfire activity is a key mechanism by which simultaneous occurrences of local PM_2.5_/ozone extremes are increasing in the western US despite declining background levels of these pollutants in response to the Clean Air Act (*23*, *32*, *56*, *57*).

Our results emphasize that atmospheric ridging patterns can affect widespread PM_2.5_/ozone co-occurrences and associated population exposure by amplifying multiple direct physical drivers and sources of air pollutants. In addition to promoting conditions that are conducive for wildfires that produce multiple harmful air pollutants, persistent ridging results in widespread heat and air stagnation that enhances ozone production. We identify a significant relationship between the extent of heat and local PM_2.5_/ozone co-occurrences. Further emphasizing the importance of meteorology in influencing population exposure to widespread air pollution conditions, large-scale airflow around high pressure ridges can transport smoke and associated pollutants to remote areas. The presence of these favorable meteorological conditions was critical in shaping the 2020 widespread co-occurrence episode via record heat and atmospheric patterns conducive to smoke transport.

The increasing frequency and persistence of ridging during the late-summer wildfire season (Fig. 4) suggest an increased likelihood of the type of atmospheric conditions that contributed to the August 2020 co-occurrence episode, if these trends continue. While recent studies have shown an intensification of western US summer ridging since the 1980s using atmospheric reanalysis (*58*) and tree-ring records (*59*), identification of trends in ridging frequency and persistence over the western US before the present analysis had been restricted to other seasons (*60*–*62*). Our findings of changing late-summer atmospheric patterns agree with recent studies that have highlighted the role of increasingly warmer and drier summer seasons, which are strongly favored by atmospheric ridging, across the western US in driving increased wildfire burned area extent and severity (*38*, *39*). Furthermore, drought and extreme heat events associated with persistent ridging can produce widespread dust and photochemical pollution-related health impacts across the western US (*63*, *64*), increasing the likelihood of compound stressors on human health.

### Limitations

We note multiple caveats to our findings. First, the derived gridded datasets of PM_2.5_ and ozone used in this study are based on a relatively sparse observational network in some parts of the western US, which might result in uncertainties in identified trends in these areas. Enhancing spatial coverage of the monitoring network is critical to get more accurate and finer-scale air quality information, particularly over rural areas of the western US. While the PurpleAir network is rapidly enhancing the PM_2.5_ observational coverage (*65*), it has notable measurement biases, and a similar low-cost network is not currently available for ozone. Second, we mainly focus on identifying proximal relationships, do not directly link wildfire emissions with local PM_2.5_/ozone co-occurrences, and do not examine the dependence of pollutant and precursor concentrations on burn severity or types of fuel burned in different landscapes. Although we explicitly link the presence of wildfire smoke to local PM_2.5_/ozone co-occurrences during the widespread episode of August 2020 using the NOAA HMS product, we do not systematically quantify the climatology of pollutant co-occurrences with or without presence of wildfire smoke due to the limited record and do not link all individual fires to pollutant co-occurrences. Last, we investigate the relationship between the extent of PM_2.5_/ozone co-occurrences and two main drivers—widespread heat and wildfire burned area—without explicitly accounting for hot, dry weather promoting further wildfires, leading to enhanced co-occurrence extent. Hot temperatures are a common underlying driver of both wildfire activity and ozone production across the western US on different time scales (*23*), and high-resolution modeling would be required to disentangle the individual contributions of heat and wildfire smoke to local PM_2.5_/ozone co-occurrences across this region.

### Implications

In recent years, millions of people across the western US have been affected by hazardous air quality conditions caused by wildfire smoke. Although PM_2.5_ concentrations are greatest in dense smoke plumes near wildfires, we find an increase in local PM_2.5_/ozone co-occurrences over widespread areas of the western US not limited to the immediate proximity of active fires. These results highlight the potential for increasing population exposure to compounding human health stressors in fire-prone and remote regions, with projected increases in wildfire activity, smoke, and conducive meteorological conditions (*51*, *66*, *67*). Although more research is needed to assess the cumulative health outcomes of co-occurrences of PM_2.5_/ozone extremes and other pollutants in wildfire smoke, it is very likely that these co-occurring air pollution extremes have compounding public health impacts (*29*). Their impacts are not only limited to the direct cardiovascular and pulmonary effects but also extend indirectly to physical and mental health consequences arising from disruptions to outdoor activity, exercise, and normal social activities. Vulnerable communities in the western US that have limited access to health care or other resources needed to cope with poor air quality, have livelihoods that involve higher occupational exposure to polluted outdoor air, or have high rates of prevalence of medical conditions that can exacerbate the effects of air pollution exposure are likely to face increasing threats from such co-stressors. Understanding the likelihood and drivers of these co-occurring hazards is, therefore, critical for protecting communities through improved planning and management of human health impacts from projected warming, drying, and increasing wildfire activity in the western US.

## MATERIALS AND METHODS

### Datasets

We use 1° × 1° gridded PM_2.5_ and ozone datasets spanning 2000 to 2020 for the United States developed using the methods of Schnell *et al.* (*46*) and subset to the western US domain (125°W to 103°W, 31°N to 49°N). These gridded datasets are derived from surface monitoring station data provided by the U.S. EPA’s Air Quality System [AQS (www.epa.gov/aqs); for PM_2.5_ and ozone], Canada’s National Air Pollution Surveillance Program (https://open.canada.ca/data/en/dataset/1b36a356-defd-4813-acea-47bc3abd859b; for PM_2.5_ and ozone), and the Clean Air Status and Trends Network (www.epa.gov/castnet; for ozone). Validated AQS data are used for PM_2.5_/ozone spanning October 2000 to July 2019, with preliminary data sourced from the AirNow online portal (www.airnow.gov) for August 2019 to September 2020. We use daily averages for PM_2.5_ and the maximum daily 8-hour average (MDA8) for ozone, reflecting the measures typically used for regulatory purposes and health impacts. For ozone, the hourly measurements are interpolated and MDA8 is calculated. For PM_2.5_, daily averages are constructed before interpolation from any hourly reporting stations, and the daily average values are interpolated. The interpolation procedure is a hybrid inverse distance-weighted method that includes a declustering component designed to limit the influence of multiple clustered, typically urban observations. Parameters for the interpolation were optimized with a leave *N*-out cross-validation procedure. These gridded datasets were originally developed for the purpose of evaluating global chemistry models for their ability to simulate large-scale, multiday air pollution episodes. They have also been used to analyze large-scale PM_2.5_, ozone, and extreme temperature co-occurrences in the eastern US (*45*); thus, they are well-suited for similar analysis of PM_2.5_/ozone co-occurrences across a large geographic region here. PM_2.5_/ozone data are analyzed over two seasons—July to September of the given year and October of the previous calendar year through June of the given year.

Meteorological data, consisting of 500-hPa geopotential heights and 2-m air temperature, were obtained from the ECMWF ERA5 reanalysis (www.ecmwf.int/en/forecasts/datasets/reanalysis-datasets/era5) on the native 0.25° × 0.25° resolution (*68*, *69*). For analyzing the colocation of wildfire smoke and PM_2.5_/ozone co-occurrence during the August 2020 case study, daily wildfire smoke polygons for 15 to 29 August 2020 were obtained from NOAA’s National Environmental Satellite, Data, and Information Service HMS smoke product (www.ospo.noaa.gov/Products/land/hms.html#data) (*19*, *54*). For each day, all polygons representing smoke of any density were merged into a single polygon representing total smoke coverage for that day (*19*) and were overlaid with the 1° × 1° grid of the PM_2.5_ and ozone datasets. Any grid cell spatially colocated with any portion of a smoke polygon is categorized as experiencing a “smoke-day,” enabling the computation of the total number of smoke-days during the 15-day episode in each grid cell. For visualization in Fig. 5B, the gridded values of smoke-day frequencies were interpolated to contours and smoothed with a Gaussian filter (σ = 0.2), allowing for the preservation of large-scale spatial features of smoke-day counts while minimizing visual noise induced by local-scale variation.

The Moderate Resolution Imaging Spectroradiometer (MODIS) Aqua+Terra Thermal Anomalies/Fire Locations 1-km dataset (MCD14DL) was retrieved from NASA’s Fire Information for Resource Management System archive download portal (https://firms.modaps.eosdis.nasa.gov/download/). The MCD14DL product is used to identify the presence of wildfires (>95% confidence) in at least 50 1-km grid cells contained within each of the larger 1° × 1° grid cells during the August 2020 widespread co-occurrence episode. The 50-km^2^ threshold was chosen to isolate large fire occurrences (*70*), as these fires are presumed to impact air quality on regional scales. To quantify the spatial extent of burned area in the western US and adjacent southwest Canada (Canadian data subset to <60°N, >115°W), we use the MODIS burned area product (2003 to 2020) (*71*).

We quantify population exposure to PM_2.5_/ozone co-occurrences using estimated 2020 population counts from the Gridded Population of the World version 4 dataset, obtained on a 1° × 1° grid from Columbia University’s Socioeconomic Data and Applications Center (https://sedac.ciesin.columbia.edu) (*72*). Western US population is defined as the total population contained in all grid cells (*n* = 375) within the study domain, which includes adjacent parts of the Great Plains and Mexico. We use person-days as a metric to quantify population exposure to local PM_2.5_/ozone co-occurrence. It is obtained by multiplying the estimated 2020 population in each grid cell by the number of co-occurrences in that grid cell and then aggregating it across the domain. We consider a fixed population to isolate the influence of changing physical hazards on changing exposure.

### Defining PM_2.5_/ozone co-occurrences

We seek to understand changes in simultaneous occurrence of extreme PM_2.5_ and ozone concentrations, as co-occurrences of both pollutants have the potential to induce co-stressor effects on human and environmental health. We therefore define extremes for PM_2.5_ and ozone at each grid cell individually as the exceedances of the local 90th percentiles of their daily concentrations (average daily value for PM_2.5_ and MDA8 for ozone) within each individual year. Therefore, we examine the co-occurrence of the top ~37 PM_2.5_ and ozone extremes in each grid cell for each year. Instead of a fixed threshold to define extremes over the study period, this time-varying definition allows us to identify extremes relative to the overall improving air pollution due to emission reductions and stricter national air quality standards. Furthermore, having a fixed number of individual occurrences of both pollutants in each year enables us to identify years with anomalous temporal co-occurrences driven by factors other than their climatology. Assuming independent distributions, in a given grid cell the joint probability of PM_2.5_/ozone co-occurrence each with a 10% chance of occurrence is 3.65 days/year, if co-occurrences are truly random. However, nearly 86% of western US grid cells have a higher likelihood of co-occurrence relative to random chance alone (fig. S6), suggesting the role of common physical drivers of such co-occurrences.

### Characterizing large-scale atmospheric patterns

To investigate the influence of large-scale atmospheric patterns on local PM_2.5_/ozone co-occurrences, we use SOMs to cluster daily geopotential height anomalies during July to September, 1979 to 2020, and identify typical atmospheric circulation patterns. SOMs are a type of artificial neural network commonly used in the climate sciences for spatial clustering of large-scale meteorological variables based on their similarity (*48*). The number and arrangement of SOM nodes are subjective choices and depend on the application (*49*, *60*). We test three SOM node configurations comprising 6 (2 × 3), 12 (3 × 4), and 20 (4 × 5) nodes to identify a configuration that minimizes similarity between clusters while also capturing the range of patterns that occur in this region.

To help inform our SOM configuration selection, we examined two sets of spatial correlation coefficients following Gibson *et al.* (*73*): (i) between each SOM node pattern and the individual constituent patterns in that node (“node-field” correlation, higher values are optimal) and (ii) between every unique combination of node pairs (“node-node” correlation, lower values are optimal). See fig. S7 for the distribution of both sets of correlation coefficients. We selected the 12-node (3 × 4) SOM configuration as the median node-field correlation is higher than in the 6-node configuration, and the node-node correlation interquartile range is lower than in the 20-node configuration. The improvement in node-field correlation in the 20-node configuration is small (fig. S7A), and this configuration qualitatively exhibits overlapping patterns due to the larger number of nodes. While the six-node configuration does have a larger distinction among nodes (based on lower median; see fig. S7B), it does not adequately represent the range of geopotential height patterns seen in the 12-node configuration. For SOM training, we use 200 initial iterations and 800 final iterations and set the initial neighborhood radius to 3 with a final neighborhood radius of 1. SOM computation was performed using the MATLAB “SOM Toolbox.”

### Examining relationships between wildfires, extreme heat, and co-occurrence extent

Local co-occurrences of PM_2.5_ and ozone extremes are a result of complex interactions between meteorology and wildfire smoke operating on multiple timescales. Our a priori assumption is that long-range transport of wildfire smoke can take several days to cover a large geographic extent of the western US. Furthermore, our hypothesis is that multiday heat waves can influence co-occurrence extent through both promoting wildfire activity that can produce air pollutants in following days and through widespread photochemical production and accumulation of ozone. To account for these interactions, we examine the relationship between antecedent fire and heat conditions in the preceding week (7-day window) with local PM_2.5_/ozone co-occurrence extent on a given day. We estimate the correlation between wildfire burned areas preceding peak co-occurrence extent and between the spatial extents of positive daily maximum temperature anomalies preceding peak co-occurrence extent.

To isolate conditions antecedent to peaks in the spatial extent of widespread co-occurrence, we extract the largest co-occurrence spatial extent in nonoverlapping 15-day windows. This is done iteratively in descending order of co-occurrence extent for all July to September days during the period of overlap with burned area data (2003 to 2020). Starting with the largest spatial extent (68.5% of the western US on 24 August 2020), a 15-day window, centered on that day, is used to exclude all other days in this window, and this process is repeated for each successive lower extent provided it is outside of all previous 15-day windows. This process yields 21 widespread co-occurrence extent peaks (of 72 total widespread co-occurrence days; see fig. S2) during July to September, 2003 to 2020, that we define as temporally independent and use in the correlation analyses to examine the relationship between the extent of burned area, heat and local PM_2.5_/ozone co-occurrences.

The highest correlation between burned area and local PM_2.5_/ozone co-occurrence extent (*r* > 0.65) for these 21 peak spatial extents occurs for lags of −3 to −7 days (fig. S8, blue line), with peak correlation at −4 days (*r* = 0.74). The highest correlation between the extent of heat and co-occurrence (*r* > 0.49) occurs for lags of −6 to −13 days (fig. S8, orange line), with peak correlation at −11 days (*r* = 0.53). We note that these lags are based on a relatively small number of peak dates, and the time of peak extent of local PM_2.5_/ozone co-occurrences following heat and fire conditions can vary for individual dates. Therefore, our use of the 7-day lagged window in this analysis captures the overlapping period of high correlation of co-occurrence extent with antecedent widespread heat conditions and burned area extent while accounting for differences in the timing of individual extent peaks.
