# Supplementary material for: Increasing co-occurrence of fine particulate matter and ground-level ozone extremes in the western United States
Source: Sci Adv. 2022 Jan 5;8(1):eabi9386. doi: 10.1126/sciadv.abi9386 (PMC8730618; doi:10.1126/sciadv.abi9386)
Supplement: Supplementary file 1 — Figs. S1 to S8 Table S1 [file sciadv.abi9386_sm.pdf]

Science Advances
AAAS

Supplementary Materials for

Increasing co-occurrence of fine particulate matter and ground-level ozone extremes in the western United States

Dmitri A. Kalashnikov*, Jordan L. Schnell, John T. Abatzoglou, Daniel L. Swain, Deepti Singh

*Corresponding author. Email: dmitri.kalashnikov@wsu.edu

Published 5 January 2022, *Sci. Adv.* **8**, eabi9386 (2022)
DOI: 10.1126/sciadv.abi9386

This PDF file includes:

Figs. S1 to S8
Table S1

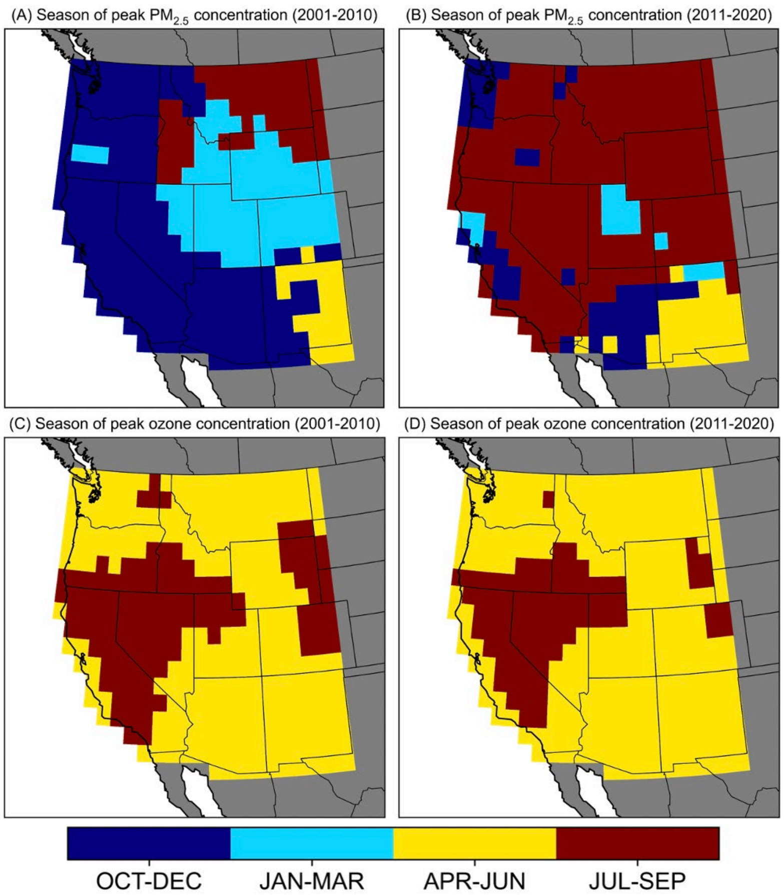

Fig. S1. Peak seasons of mean $PM_{2.5}$ and ozone concentrations. Seasons of peak average concentrations of $PM_{2.5}$ (top) and ozone (bottom) during 2001-2010 (left) and 2011-2020 (right) at each grid cell.

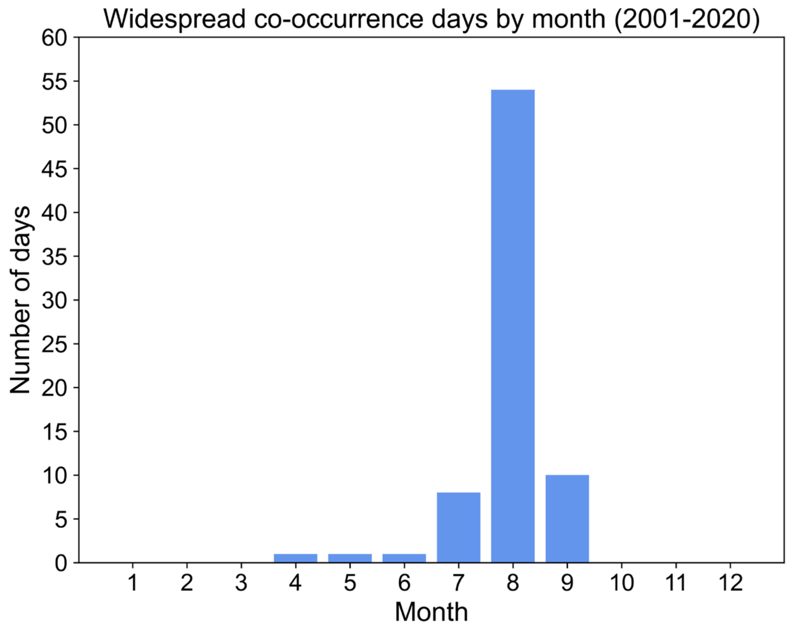

Fig. S2. Seasonality of widespread co-occurrence days. Total number of widespread $PM_{2.5}$/ozone co-occurrence days (≥25% of the western US) during each calendar month, 2001-2020.

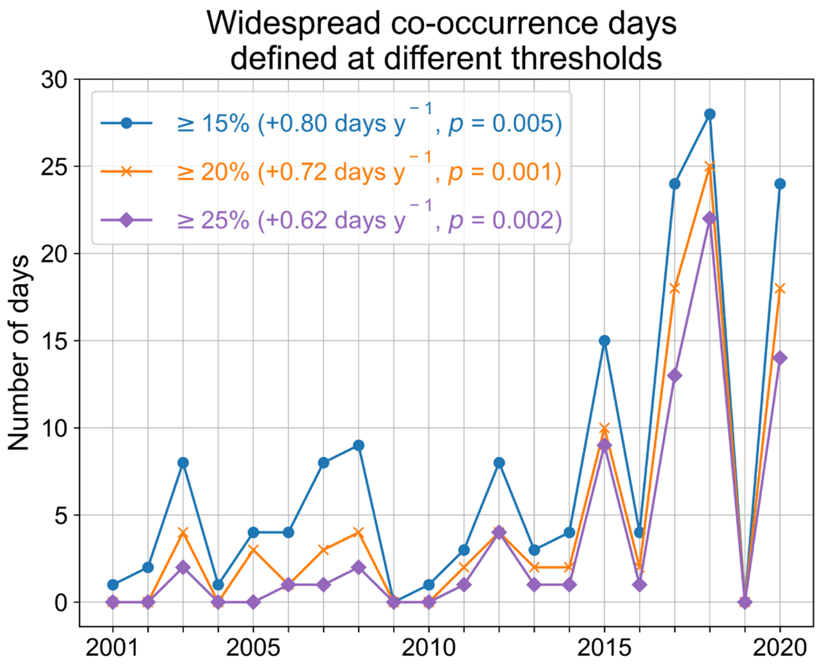

Fig. S3. Widespread $PM_{2.5}$/ozone co-occurrence days during July-September at different thresholds. Days with simultaneous local $PM_{2.5}$/ozone co-occurrences affecting ≥15% (green), ≥20% (brown), and ≥25% (blue) of western US grid cells, 2001-2020. Text indicates annual linear trends and *p*-values based on a non-parametric permutation test.

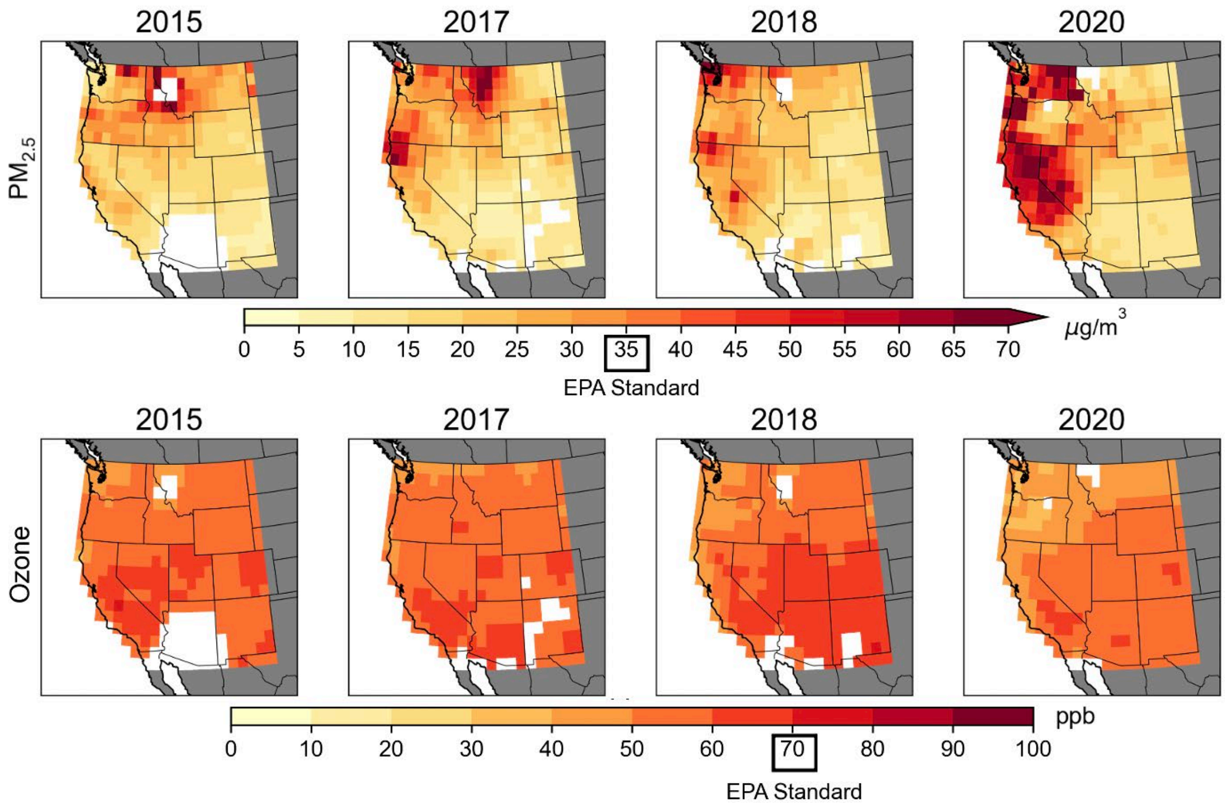

Fig. S4. $PM_{2.5}$ and ozone concentrations on co-occurrence days. Average concentrations of $PM_{2.5}$ (top row) and MDA8 ozone (bottom row) on all local co-occurrence days during July-September of 2015, 2017, 2018, and 2020. Corresponding EPA regulatory health standards are 35 μg/m^3 for $PM_{2.5}$ and 70 ppb for ozone. White shading indicates that no co-occurrence days were recorded in those grid cells during July-September of that year.

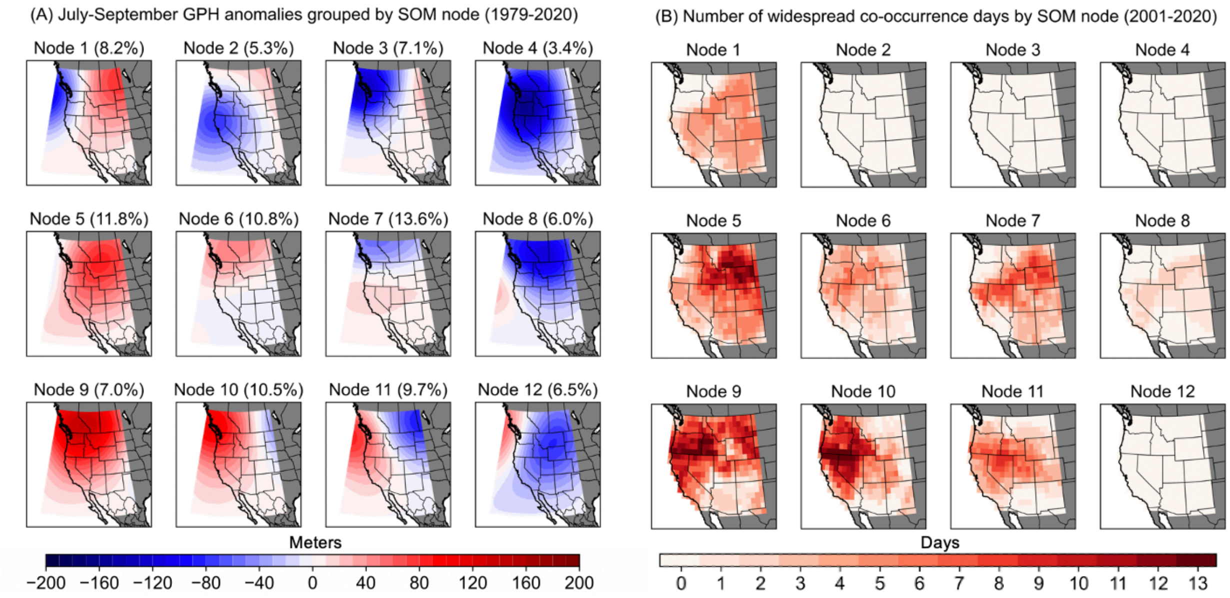

Fig. S5. All 12 nodes of the Self-Organizing Map (SOM). (**A**) Geopotential height (GPH) anomalies for each SOM node trained over 1979-2020. (**B**) Number of widespread $PM_{2.5}$/ozone co-occurrence days (2001-2020) associated with each node. Values in parentheses in plot (**A**) indicate the frequency of each SOM node relative to all July-September days during the period of overlap with air pollution data (2001-2020).

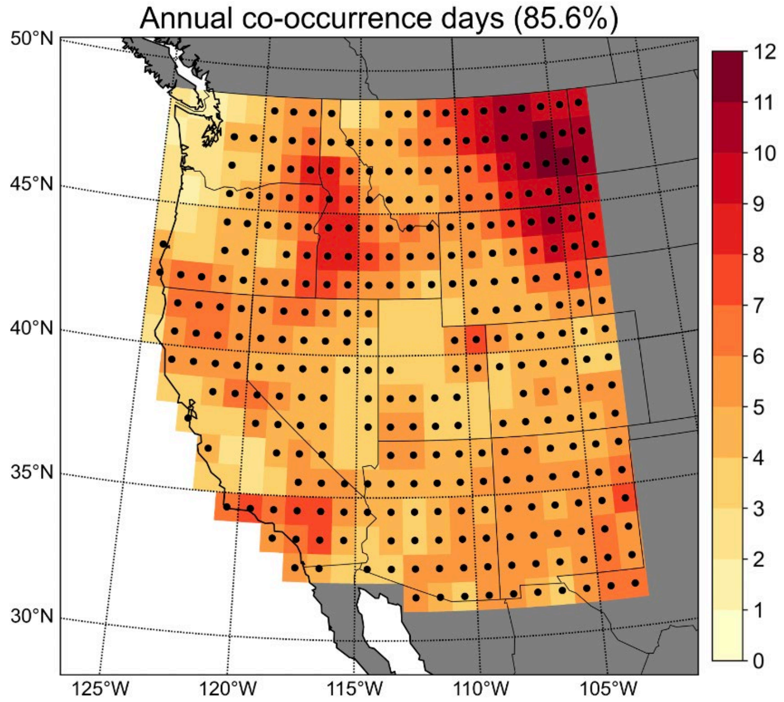

Fig. S6. Annual frequency of co-occurrence days. Average number of local annual $PM_{2.5}$/ozone co-occurrence days at each grid cell. Black dots denote grid cells averaging more than 3.65 days/year, the number expected by random chance from a joint probability distribution. Value in parentheses indicates the percentage of western US grid cells averaging more co-occurrence days than expected by random chance.

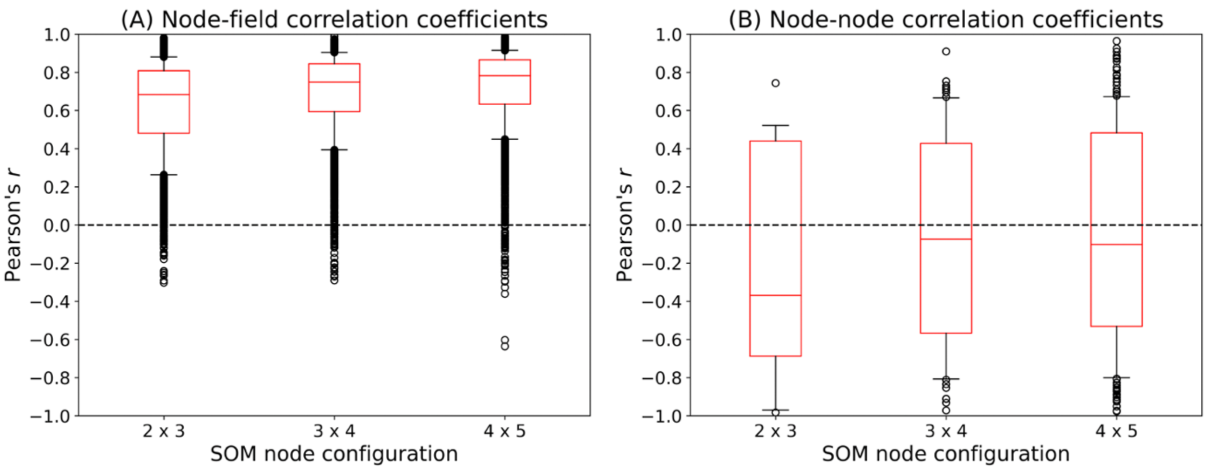

Fig. S7. Spatial correlation coefficients for three SOM node configurations. Correlation coefficients (**A**) between each SOM node pattern and the individual constituent patterns in that node, and (**B**) between every unique combination of node pairs. Higher correlation coefficients in plot (**A**) indicate that individual days are well-represented by the node pattern into which they are assigned, and in plot (**B**) indicate greater redundancy of nodes in the SOM.

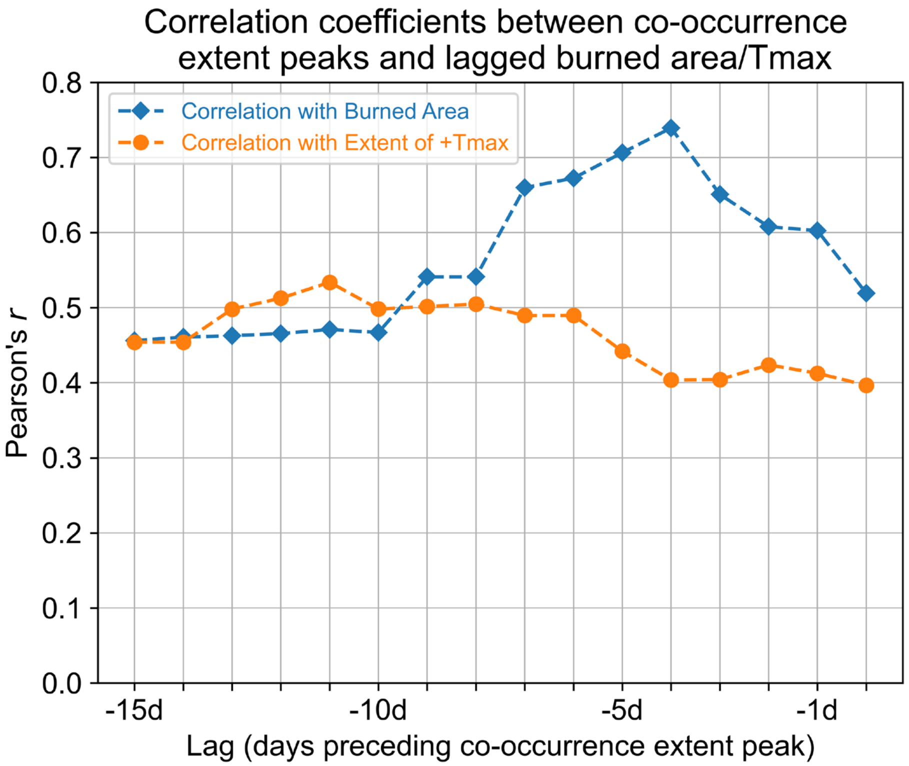

Fig. S8. Lagged relationship widespread co-occurrences, burned area, and maximum temperatures. 7-day lagged correlation coefficients between temporally independent peaks in widespread $PM_{2.5}$/ozone co-occurrence spatial extent (≥25% of western US, $n = 21$) and peaks in daily burned area in the western US and southwest Canada (blue dashes), and between peaks in widespread co-occurrence extent and peaks in the extent of positive maximum temperature (+Tmax) anomalies >1 standard deviation above local daily climatologies in the western US (orange dashes) for all lags between 0-15 days preceding $PM_{2.5}$/ozone co-occurrence.

Table S1. Top 15 days with the largest extent of local $PM_{2.5}$/ozone co-occurrences in the western US (2001-2020).

Rank	Date	Percent of western US grid cells	Population exposed, in millions
**1**	2020-08-24	68.5	36.7
**2**	2020-08-22	67.5	42.6
**3**	2020-08-25	66.4	27.9
**4**	2020-08-21	66.1	46.3
**5**	2020-08-23	64.3	36.7
**6**	2018-08-02	56.0	19.7
**7**	2018-08-01	54.4	20.4
**8**	2018-08-10	53.9	19.5
**9**	2018-08-09	51.7	35.5
**10**	2020-08-26	50.9	22.0
**11**	2015-08-20	50.7	30.1
**12**	2020-08-20	50.1	41.3
**13**	2017-09-02	46.9	42.4
**14**	2015-08-21	46.9	25.8
**15**	2018-08-08	46.1	34.7
